# Transfer learning for drug–target interaction prediction

**DOI:** 10.1093/bioinformatics/btad234

**Published:** 2023-06-30

**Authors:** Alperen Dalkıran, Ahmet Atakan, Ahmet S Rifaioğlu, Maria J Martin, Rengül Çetin Atalay, Aybar C Acar, Tunca Doğan, Volkan Atalay

**Affiliations:** Department of Computer Engineering, Middle East Technical University, Ankara 06800, Turkey; Department of Computer Engineering, Adana Alparslan Türkeş Science and Technology University, Adana 01250, Turkey; Department of Computer Engineering, Middle East Technical University, Ankara 06800, Turkey; Department of Computer Engineering, Erzincan Binali Yıldırım University, Erzincan 24002, Turkey; Department of Computer Engineering, Iskenderun Technical University, Hatay 31200, Turkey; Faculty of Medicine, Institute for Computational Biomedicine, Heidelberg University and Heidelberg University Hospital, Heidelberg 69120, Germany; European Molecular Biology Laboratory, European Bioinformatics Institute (EMBL–EBI), Cambridge, Hinxton CB10 1SD, United Kingdom; Faculty of Pulmonary and Critical Care Medicine, the University of Chicago, Chicago, IL, 60637, United States; Cancer Systems Biology Laboratory (Kansil), Middle East Technical University, Ankara 06800, Turkey; European Molecular Biology Laboratory, European Bioinformatics Institute (EMBL–EBI), Cambridge, Hinxton CB10 1SD, United Kingdom; Department of Computer Engineering, Hacettepe University, Ankara 06800, Turkey; Department of Computer Engineering, Middle East Technical University, Ankara 06800, Turkey

## Abstract

**Motivation:**

Utilizing AI-driven approaches for drug–target interaction (DTI) prediction require large volumes of training data which are not available for the majority of target proteins. In this study, we investigate the use of deep transfer learning for the prediction of interactions between drug candidate compounds and understudied target proteins with scarce training data. The idea here is to first train a deep neural network classifier with a generalized source training dataset of large size and then to reuse this pre-trained neural network as an initial configuration for re-training/fine-tuning purposes with a small-sized specialized target training dataset. To explore this idea, we selected six protein families that have critical importance in biomedicine: kinases, G-protein-coupled receptors (GPCRs), ion channels, nuclear receptors, proteases, and transporters. In two independent experiments, the protein families of transporters and nuclear receptors were individually set as the target datasets, while the remaining five families were used as the source datasets. Several size-based target family training datasets were formed in a controlled manner to assess the benefit provided by the transfer learning approach.

**Results:**

Here, we present a systematic evaluation of our approach by pre-training a feed-forward neural network with source training datasets and applying different modes of transfer learning from the pre-trained source network to a target dataset. The performance of deep transfer learning is evaluated and compared with that of training the same deep neural network from scratch. We found that when the training dataset contains fewer than 100 compounds, transfer learning outperforms the conventional strategy of training the system from scratch, suggesting that transfer learning is advantageous for predicting binders to under-studied targets.

**Availability and implementation:**

The source code and datasets are available at https://github.com/cansyl/TransferLearning4DTI. Our web-based service containing the ready-to-use pre-trained models is accessible at https://tl4dti.kansil.org.

## 1 Introduction

Drugs are chemical compounds that are used to treat diseases or to increase the quality of life. A drug is intended to interact with a target biomolecule (e.g. a single protein, several proteins or a protein complex) by regulating or correcting cellular functions in pathological conditions. Although drug discovery is traditionally a long, laborious, and costly process, recently, there have been innovative and promising computational solutions based on machine learning and deep learning. Virtual screening of compounds against a target cell or protein is used widely during the initial steps of the drug discovery process. Lately, deep learning-based models for virtual screening and drug–target interaction (DTI) prediction have yielded highly promising results (Chen et al. 2018; Jing et al. 2018; Lo et al. 2018; Wang and Kurgan 2018; Ezzat et al. 2019; Vamathevan et al. 2019; Baskin 2020; Réda et al. 2020; Rifaioglu et al. 2020; Bagherian et al. 2021; Elbadawi et al. 2021; Kim et al. 2021; Du et al. 2022; Pan et al. 2022; Zhang et al. 2022). However, the majority of deep learning models developed thus far require a large volume of training data. Such a large volume of data is not available for many of the target proteins or protein families, and therefore, no prediction models are available for these classes of proteins.

**Figure 1.**
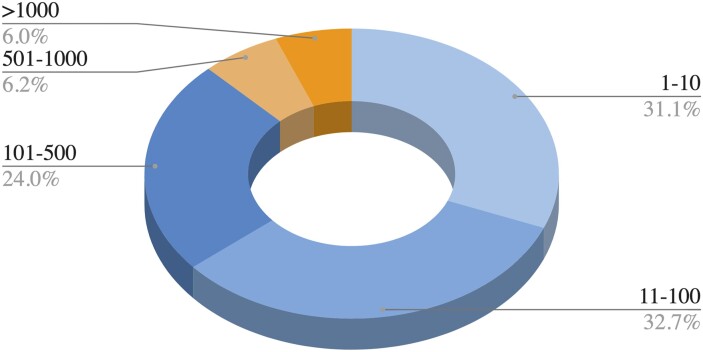
The percentage of target proteins with certain numbers of bioactive compounds in the ChEMBL_29 database (data filters: targets are single proteins and belong to the human, bioactivities are associated with a pChEMBL value, all data points are coming from binding assays, and multiple bioactivity data points for the same compound-target pair are counted as one). For example, 6.2% of the target proteins in ChEMBL have bioactive compounds in the range between 501 and 1000.

The distribution of the number of bioactive compounds per target protein in the ChEMBL_29 (Gaulton et al. 2012) database (i.e., ChEMBL database version 29) reveals the problem of limited training data. Fig. 1 shows the distribution of target proteins (percentage) in the bins of number of bioactive compounds in ChEMBL_29. It is observed from Figure 1 that nearly the two third of all human target proteins have less than 100 known bioactive compounds and nearly the half of this have less than 10 known bioactive compounds. In this case, we ask the following question: is DTI prediction possible when a limited amount of bioactivity data is available for a target protein? The crucial issue is to find a solution when a protein (or a protein family) has a low amount of bioactivity data, particularly in the case where there is the risk of overfitting for the prediction model since the input feature vector is generally of high dimensionality and deep learning models are prone to memorizing rather than learning without sufficient training data. When transfer learning or few- or zero-shot learning is incorporated, it becomes possible to learn from such a low amount of data. Transfer learning is a machine learning approach where a model is trained for a source task, and this pre-trained source model is then reused as an initial configuration to build (train) a model (target model) for a different but related target task. The basic principles and methods of transfer learning in deep artificial neural networks are explained by (Yosinski et al. 2014), while (Tan et al. 2018) have compiled studies using deep transfer learning.

Deep transfer learning has not been extensively exploited so far in the area of DTI prediction (Lee et al. 2019; Cai et al., 2021; Playe and Stoven 2020; Kao et al. 2021; Li et al. 2021). To this end, we investigate the use of deep transfer learning for the prediction of interactions between drugs/compounds and understudied target proteins that have scarce training data and we present a systematic evaluation for this aim. We formulated DTI prediction as a ligand-based binary classification problem. For this, a feed-forward neural network (FNN) with two hidden layers is used in which the input is a 300-dimensional vector representation of a compound and the output is a binary value that indicates whether the given input compound is predicted to interact with the target family. In terms of data, we have selected six of the main protein families: G-protein-coupled receptors (GPCRs), ion channels, kinases, nuclear receptors, proteases, and transporters. Transporters and nuclear receptors were separately used as the target dataset, while the other five families were set as the source datasets. Deep transfer learning was carried out by training the FNN with the experimental bioactivity measurements of a source dataset generated from one of the five protein families and applying the three modes of transfer learning on the small-sized target family (transporter or nuclear receptor) training datasets. The small-sized target family training datasets are generated in a controlled manner to pursue a systematic evaluation approach. We then compared the performance of this deep transfer learning approach with the case where the FNN was trained from scratch. We also compared it against a shallow classifier.

In Section 2, we give background information on deep transfer learning. This is followed by a discussion of the related research in Section 3. The data (Section 4) and method (Section 5) are then presented in addition to the experimental evaluation (Section 6). Section 7 finally presents a discussion and conclusions.

## 2 Background information-deep transfer learning

A machine learning problem involves a domain, *D*, and a task, *T*. Given a source problem and a target problem, the source domain is *D_s_*, and the target domain is called *D_t_*, while the source task is *T_s_*, and the target task is *T_t_*. Transfer learning aims to learn *D_t_* and improve the performance of *T_t_* with the help of *D_s_* and *T_s_*. In practice, a domain is represented by a dataset. During the initial steps of drug discovery, the task is typically the prediction of the interaction or bioactivity of the drug with the target protein(s) or the prediction of the absorption, distribution, metabolism, elimination, and toxicity of the drug. The domain is typically the set of molecules described by features such as chemical descriptors. In our case, the task remains the same, and the transfer is between domains, i.e. between different molecular (compound) datasets.

Deep transfer learning is applying transfer learning on deep neural networks. The training phase of deep transfer learning is composed of two stages.

*Stage I*: A source model is obtained by training the network with a sufficient number of source training data. This is also referred to as the pre-trained source model.

*Stage II*: The pre-trained source model is used as an initial configuration and re-trained using target training data (which is typically small) to obtain a target model.

Techniques for Stage II are grouped under three modes. Note that the architecture of a deep neural network can be functionally decomposed into roughly two parts: the bottom layer(s) where feature extraction is performed and the upper layer(s) where prediction is performed. Mode 2 and Mode 3 make use of this functional decomposition of the network.

*Mode 1—Full fine-tuning*: The most common deep transfer learning technique is fine-tuning, which is in fact parameter-based transfer learning. Based on the assumption that the learned parameter values (weights) contain useful knowledge obtained from the source domain, we seek to achieve better performance by moving these parameter values (weights) to the target model. The parameter values acquired from the source model form the initial values of the parameters of the target model. In this way, the weights of the target model do not start with random values but with the converged values of the weights of the pre-trained source model, and the target model is re-trained with a small number of target training data and converges faster as well with a reduced number of training epochs (Fig. 3a).

*Mode 2—Feature transformer*: The source model is in fact used to form a latent feature space i.e. common to both source data and target data. This is indeed feature-based transfer learning. The feature transformer can be obtained by freezing the bottom layers (which are used for feature extraction) of the pre-trained source model during Stage II; i.e. the weights of the nodes at the bottom layers are not updated during re-training with the target training data. Only the weights of the nodes at the output layer (i.e. the predictor) are modified with the limited number of target training data (Fig. 3b).

*Mode 3—Shallow classifier*: In Stage II, the output layer (predictor) of the source model is replaced with a shallow classifier. Hence, only the shallow classifier is trained with the target data and the feature vectors for the target data are extracted by the frozen bottom layers of the source model. Mode 3 is similar to Mode 2, except that the extracted feature vectors are given to a shallow classifier instead of the output layer (predictor) of the neural network model (Fig. 3c).

## 3 Related research

A comprehensive literature review on transfer learning in drug discovery is given by (Cai et al., 2021). In the drug discovery field, most deep transfer learning studies have been carried out for the prediction of compound properties, generation of molecules, and structure-based virtual screening. Here, we focus on deep transfer learning studies related to ligand-based and feature-based chemogenomic DTI prediction methods. Multi-channel PINN (Lee et al. 2019), a pairwise-input neural network model (chemogenomic DTI predictor) was trained for the classification of compound–target protein interaction, and toxicity (activity) was chosen as the target task in transfer learning. When compared, transfer learning was more successful than training from scratch. MPG (Li et al. 2021) employed graph neural networks with the aim of compound representation learning and by using this GNN as a feature transformer, a chemogenomic DTI predictor was assessed. The study was not necessarily carried out on limited data or small datasets. EnsembleDLM (Kao et al. 2021) made an extensive analysis and examined how much data a network (chemogenomic DTI regressor) needs to achieve an acceptable DTI prediction performance via transfer learning with full fine-tuning through several datasets, including KIBA, Davis, and some others extracted from ChEMBL. The main critical point of this study is that most of these datasets are from the same protein family, i.e. enzymes. Another comprehensive study on transfer learning in DTI prediction (chemogenomic DTI binary classifier) is by Playe and Stoven (2020). They reported that transfer learning by full fine-tuning technique might improve the prediction performance if the source task is highly similar to the target task. Dey et al. used instance-based and feature-based transfer learning in contrast to the popular parameter-based transfer learning, such as pre-training (Dey et al. 2022). Yang et al. employ transfer learning to predict protein–protein interactions (Yang et al. 2021). Our study differs majorly in two ways. First, Yang et al. did not consider the case of having limited data for prediction. Second, Yang et al. solely employed Mode 1 and Mode 2 methods of our transfer learning methods, while neglecting the use of Mode 3.

## 4 Data

Training datasets and test datasets were generated from the ChEMBL database version 29 by applying the data filtering protocol developed in our previous study (Rifaioglu et al. 2020). pChEMBL value = 7.0 (XC50 = 100 nM) was used to separate the active and inactive compounds of each target. Data points in the dataset were filtered based on certain attributes such as “target type” (i.e. single protein), “taxonomy” (i.e. human), “assay type” (i.e. binding and functional assays), and “standard type” (i.e. IC50, EC50, AC50, Ki, Kd, and Potency). There were some duplicate measurements in the dataset that originated from different bioassays. To handle this, the median bioactivity value was identified for each pair and assigned as the sole bioactivity measurement. To avoid any ambiguity related to the physical binding of compounds to their targets, the functional assays were discarded and only the binding assays were kept after additional filtering based on “assay type”. Finally, bioactivity measurements without a pChEMBL value were removed from the dataset.

The compounds were converted into ECFP4 fingerprints and then clustered using RDkit’s compound clustering function (Butina 1999) based on a Tanimoto coefficient value of 0.8. This was done to avoid bias toward specific chemical series when training and evaluating the model. The statistics for the datasets after the filtering steps are given in Table 1. To construct source training datasets, target training datasets, and test datasets, we have chosen six main protein families: GPCRs, ion channel, kinase, nuclear receptor, protease, and transporter. The transporter and nuclear receptor families were selected as the target datasets (separately), while the other five families were used as the source datasets.

**Table 1.** Numbers of active and inactive compounds, training dataset size, and test dataset size for all protein families.

Protein family	Active	Inactive	Training dataset	Test dataset
GPCR	36, 924	31,085	56, 675	11, 334
Ion channel	5,996	14, 167	16, 803	3,360
Kinase	35 ,531	30, 778	55, 259	11, 050
Nuclear receptor	5,099	6,668	9,807	1,960
Protease	15, 718	19, 518	29 ,364	5,872
Transporter	3,666	5,898	7,970	1,594

To generate training datasets containing lower numbers of DTI data points in a controlled manner, we randomly picked compounds from the original transporter training dataset and nuclear receptor training dataset. Eight smaller and balanced (containing the same number of active and inactive compounds) target training datasets were constructed where the numbers of bioactivities are 2, 6, 12, 48, 96, 400, 1000, and 4000. Tests for all eight smaller target training datasets were carried out with the transporter family test dataset (containing 1594 bioactivity data points in the test dataset) and the nuclear receptor family test dataset (containing 1960 bioactivity data points in the test dataset).

## 5 Method

We formulated DTI prediction as a ligand-based binary classification problem. Therefore, we considered deep neural networks having compound features at the input which perform binary classification (i.e. having binary output).

**Figure 2.**
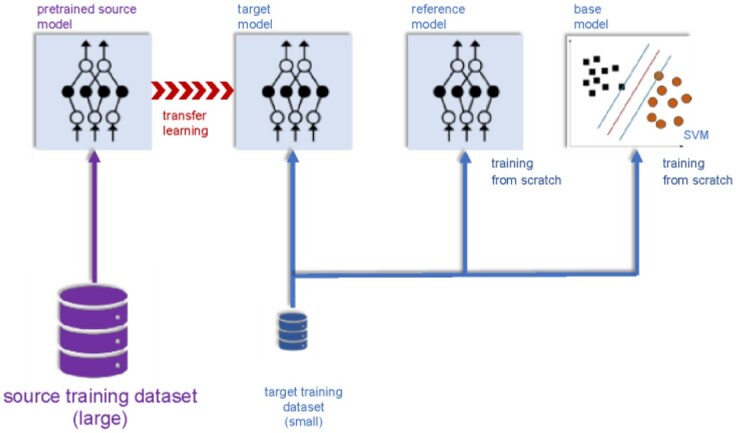
Sketch of the training phase. During the training phase, we first trained a source neural network model with a training dataset of a source family (Stage I). This pre-trained source model is then used for transfer learning to retrain it with a small-sized target training dataset (Stage II). We also trained, from scratch, an FNN having exactly the same configuration (reference model) as well as a shallow classifier (base model), using this same target training dataset.

The training phase is sketched in Fig. 2. At the same time as the training phase, for comparison purposes, we trained, from scratch, an FNN having exactly the same configuration (reference model) as well as a shallow classifier (base model), using this same target training dataset. During the test phase, all three models trained with the same target training dataset are tested with an independent target test dataset and the performance is evaluated and comparisons are made. All datasets are generated using the learned representations of Chemprop (Yang et al. 2019).

**Figure 3.**
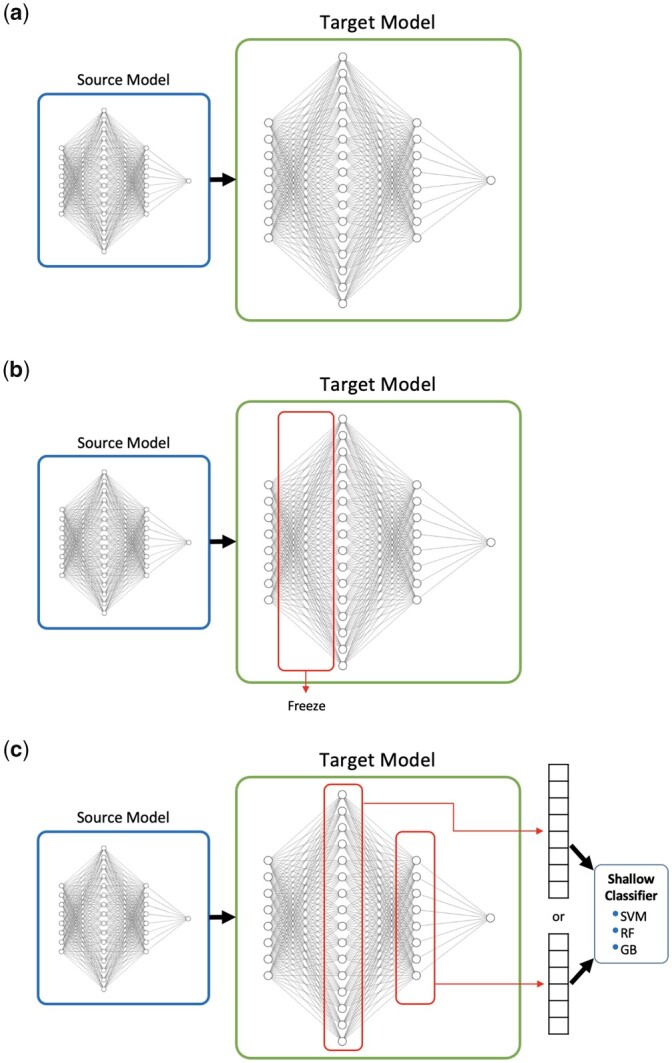
Visual representations of three modes of transfer learning (described in Section 2) on FNN-2-Chemprop; a) Mode 1: full fine-tuning, b) Mode 2: feature transformer, and c) Mode 3: shallow classifier.

To choose a neural network architecture and determine its configuration, we compared the performances of several different architectures such as FNN with various numbers of hidden layers, one-dimensional convolutional neural network (1D-CNN), two-dimensional CNN (2D-CNN) with various input compound representations. The best performance is obtained by the model composed of an FNN with two hidden layers where Chemprop learned representations (FNN-2-Chemprop) are used as input. FNN-2-Chemprop performs binary classification (as active or inactive) using compound features at the input level. In FNN-2-Chemprop, a compound is represented by a numerical vector of length 300 which is obtained by using the learned representations of Chemprop. Training and test split was employed to tune the hyperparameter values. The final values of hyperparameters used in FNN-2-Chemprop are as follows: number of hidden layers = 2; hidden layer sizes = 1200 and 300; learning rate = 0.0001; number of training epochs = 100; batch size = 256. Visual representations of three modes of transfer learning (described in Section 2) on FNN-2-Chemprop are demonstrated in Fig. 3.

Representations (features) of compounds are learned by using Chemprop (Yang et al. 2019). Chemprop is a graph CNN model consisting of two parts: a Directed Message Passing Neural Network (DMPNN) and an FFN. Message Passing Neural Network (MPNN) is a model that works on an undirected graph with node properties and edge properties. In Chemprop, training data for each compound includes the SMILES string and a target value for the task. In this study, we trained Chemprop to perform the relevant task as a binary classifier (e.g. discriminating between active and inactive compounds in the training dataset of the kinase protein family). We then removed the final classifier FNN layer and we used the values of 300 nodes in the last layer of the DMPNN as the representative (feature vector) of the compounds both in the training dataset and test dataset of the protein families. Thus, representations were learned for a specific task.

## 6 Experimental evaluation

For comparison purposes, we selected the FNN-2-Chemprop that was trained from scratch with the whole target training dataset (without any transfer learning involved) as the reference model and a Support Vector Machine (SVM) that was trained from scratch again with the whole target training dataset (without any transfer learning involved) as the base model. An SVM was used as the shallow classifier in Mode 3. XGBoost and Random Forest were also used in Mode 3. However, since SVM gave the best results among the shallow classifiers we tested, we have only indicated the results of the shallow SVM classifier.

Matthew’s correlation coefficient (MCC) was chosen as the evaluation metric to measure performance. In addition to MCC, we have already used different evaluation metrics such as AUROC, Precision, Recall, F1-score, and Accuracy which are the metrics that help to measure the robustness and stability of the models. The tables containing all the evaluation metrics are given on the GitHub page (https://github.com/cansyl/TransferLearning4DTI#results). We were able to indicate only the MCC values because of the space limitation. Furthermore, we saw that these other evaluation metrics have the same tendency as MCC over the models. The recall values are always better than the precision values for various dataset sizes and models. The difference between the recall and precision increases when the dataset size gets smaller. The high recall value means a low false negative rate, which is beneficial for DTI prediction.

**Figure 4.**
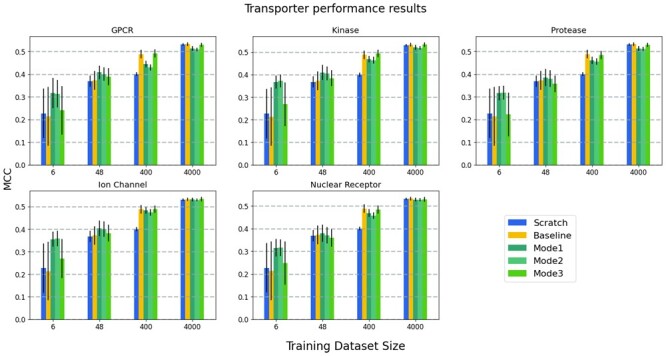
Prediction performance results of the models where transporter is the target family and one of the other five families is the source family in each panel: The average test MCC values of the reference model (FNN-2-Chemprop trained from scratch), the base model (an SVM trained from scratch) and the three modes of transfer learning. The results are given for four different cases (i.e., target training dataset sizes).

**Figure 5.**
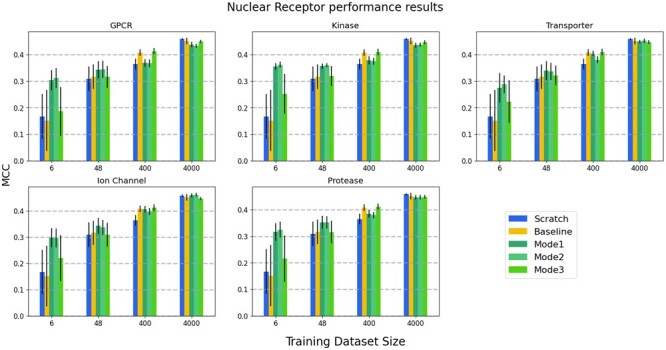
Prediction performance results of the models where nuclear receptor is the target family and one of the other five families is used as the source family in each panel: The average test MCC values of the reference model (FNN-2-Chemprop trained from scratch), the base model (an SVM trained from scratch) and the three modes of transfer learning. The results are given for four different cases (i.e., target training dataset sizes).

Average test MCC values of the reference model (FNN-2-Chemprop trained from scratch), the base (shallow) model (an SVM trained from scratch), and the three transfer learning modes are shown in Fig. 4, for four test dataset sizes. MCC values are the averages of several repeated experiments. Small datasets were created randomly for every experiment. In each case, the transporter is the target family and one of the other five families is used as the source family. A similar evaluation is given in Fig. 5 where the nuclear receptor is the target family and one of the other five families is used as the source family.

**Figure 6.**
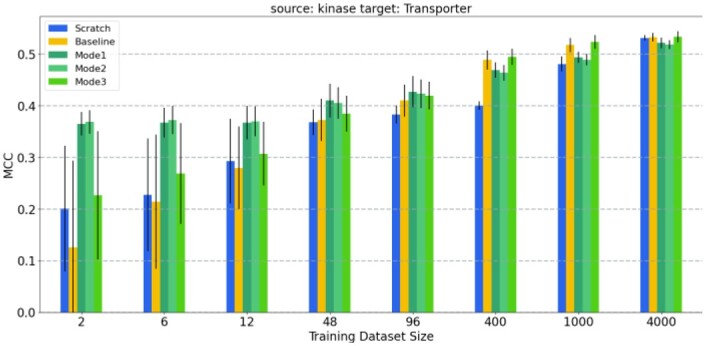
Prediction performance results of the models where transporter is the target family and kinase is the source family: The average test MCC values of the reference model (FNN-2-Chemprop trained from scratch), the base model (an SVM trained from scratch) and the three modes of transfer learning. The results are given for eight different cases (i.e., target training dataset sizes).

**Figure 7.**
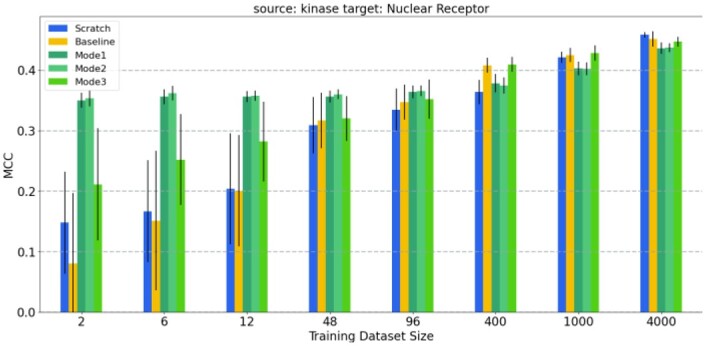
Prediction performance results of the models where nuclear receptor is the target family and kinase is the source family: The average test MCC values of the reference model (FNN-2-Chemprop trained from scratch), the base model (an SVM trained from scratch) and the three modes of transfer learning. The results are given for eight different cases (i.e., target training dataset sizes).

One of the subplots of Fig. 4, where the source protein family is kinase and the target protein family is the transporter, is given in detail in Fig. 6. The effect of learning by transfer is better understood in this plot. A similar subplot is given in Fig. 7, where the source protein family is the nuclear receptor. Similar trends occur when plots are drawn for other source protein families.

**Figure 8.**
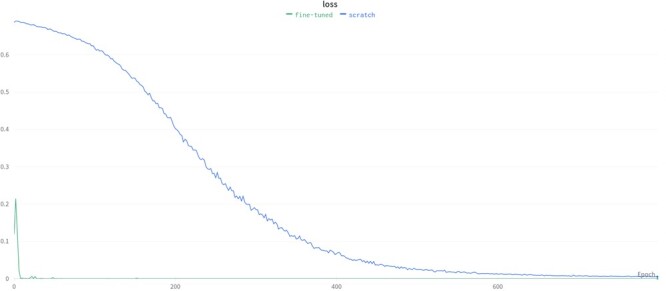
When transfer learning is used, the target (fine-tuned) models starts the training from lower loss values compared to the reference model (scratch), and converge after significantly lower number of epochs.

**Figure 9.**
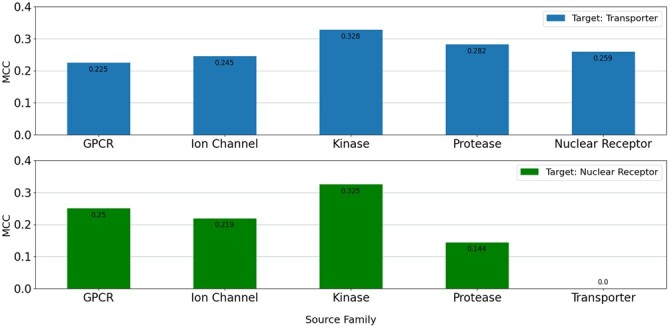
Prediction performance results of the models where the source models are directly used for inference on the target data; i.e. no transfer learning (no further training is applied on the pre-trained source model): The average test MCC values of the reference model (FNN-2-Chemprop trained from scratch) when transporter and nuclear receptor are the target families, respectively.

In general, when the training dataset size is ˂100, transfer learning has better performance than training from scratch (i.e. compared to the reference model and the base model). When the size of the target training dataset is ˃100, transfer learning performance is very close to that of training the network model from scratch. Transfer learning should still be preferred since it requires a smaller number of training epochs. In all of the cases for which the training dataset size is ˂100, transfer learning methods performed better than the reference model and base model. Furthermore, when transfer learning is used, target models start from lower loss values when compared to the reference model (see Fig. 8). Therefore, a lower number of epochs are generally sufficient for training, significantly reducing training time. We have also evaluated the performance when the source models are directly used for the tests of target data in the zero-shot learning setting; i.e. no transfer learning (no re-training is applied on the pre-trained source model). Fig. 9 shows the average test MCC values of the reference model (FNN-2-Chemprop trained from scratch) when (i) transporter and (ii) nuclear receptor are the target families, respectively. Although these results may still be acceptable, it is easily possible to increase the prediction performance via a transfer learning-based target-specific training (fine tuning) , even with a very small training dataset . For example, when the ion channel is the source family and the transporter is the target family, an MCC value of 0.333 is obtained if the pre-trained source model is trained (fine tuned) with only two samples (using Mode 1), while an MCC value of 0.245 is obtained without target-specific training.

## 7 Discussion and conclusion

Here, we present a systematic evaluation of transfer learning in DTI prediction by pre-training a neural network with source training datasets and applying different modes of transfer learning from the pre-trained source network to a target dataset by decreasing its size in a controlled manner. Working with small datasets can often pose significant challenges and require specific approachesstrategies to address issues such as overfitting or limited generalization. Yet, we found that when the training dataset is smaller than 100 compounds, transfer learning yields significantly better performance compared to training the system from scratch, suggesting an advantage to using transfer learning to predict binders to under-studied targets.

 With this approach, learning is still possible even when there is a small amount of available data, as low as only two compounds (i.e. one positive interaction data point and one negative data point corresponding to an inactive compound). Although fine-tuning is the most popular transfer learning technique, we show that the other transfer learning techniques described in this study (i.e., feature transformer and sshallow classifier) deserve attention as well. Furthermore, deep transfer learning is effective even when there are sufficient data to train a model from scratch since convergence is faster. The results table on the GitHub page (https://github.com/cansyl/TransferLearning4DTI#results) shows that Mode 3 is slightly better than the baseline model in most of the experiments, even when the number of target training examples is higher than 400. It is also possible to directly use a source model (without any fine tuning) to infer bioactive compounds for a target family dataset in the zero-shot learning setting. The performance is acceptable in this setting; however, a short target-specific fine-tuning via transfer learning (e.g., using only two target samples) boosts the performance.

The main problem that we would like to tackle next is to predict binders for a specific protein that has limited or no bioactivity data. The reason for using protein families in this study was to explore the limits (in terms of the number of bioactivity data) where transfer learning is still effective, by providing a large dataset as the source training dataset. We intend to use these family-specific models as a basis for developing target-specific models. That being said, fine tuning a target-specific model is currently possible using our pre-trained source models available in our data repository (https://github.com/cansyl/TransferLearning4DTI) and web-service (https://tl4dti.kansil.org/). Last but not least, transfer learning is not limited to DTI; the methodology presented here can be utilized for other things types of machine learning applications in biology and medicine, such as the prediction of protein functions or the effects of genomic varations.
